# Supplementary material for: Response of Mycobacterium smegmatis to the Cytochrome bcc Inhibitor Q203
Source: Int J Mol Sci. 2022 Sep 7;23(18):10331. doi: 10.3390/ijms231810331 (PMC9498996; doi:10.3390/ijms231810331)
Supplement: Supplementary file 1 [file ijms-23-10331-s001.zip › ijms-1893809-supplementary.pdf]

Supplementary file

Response of *Mycobacterium smegmatis* to the cytochrome *bcc* inhibitor Q203

Supplementary tables and figures

Table S1. Oligonucleotide primers used in the study.

Gene	Primer name	Primer sequence (5`-3`)	Purpose
16S *rRNA*	16s *rRNA* F<br>16s *rRNA* R	CTTACCTGGGTTTGACATGC<br>CTGGCAACATGAGACAAGG	qPCR analysis
qcrB	*qcrB* F<br>*qcrB* R	TGTACTTCGTCGCCTACC<br>AAGTCGCTTGATGATGCC	
cydA	*cydA* F<br>*cydA* R	GCGGTCATCTGGTTCTTCC<br>CTAGTAGGCGAACGACAACG	
appB	*appB* F<br>*appB* R	GAGGCCAACCACGTATGG<br>CGGGATGAACAGTGTTGTCG	
appC	*appC* DNA F<br>*appC* DNA R	ATGGTGTTCACCGAAACCCTGCTTCT GCTCG<br>TCATGACCGGGCCTCCGTGCG	PCR with genomic DNA
appB	*appB* DNA F<br>*appB* DNA R	ATGACGCAGGCCACCTTCGTCGC<br>TCAGCGTGACCACTCCTCGGTCTGCG	

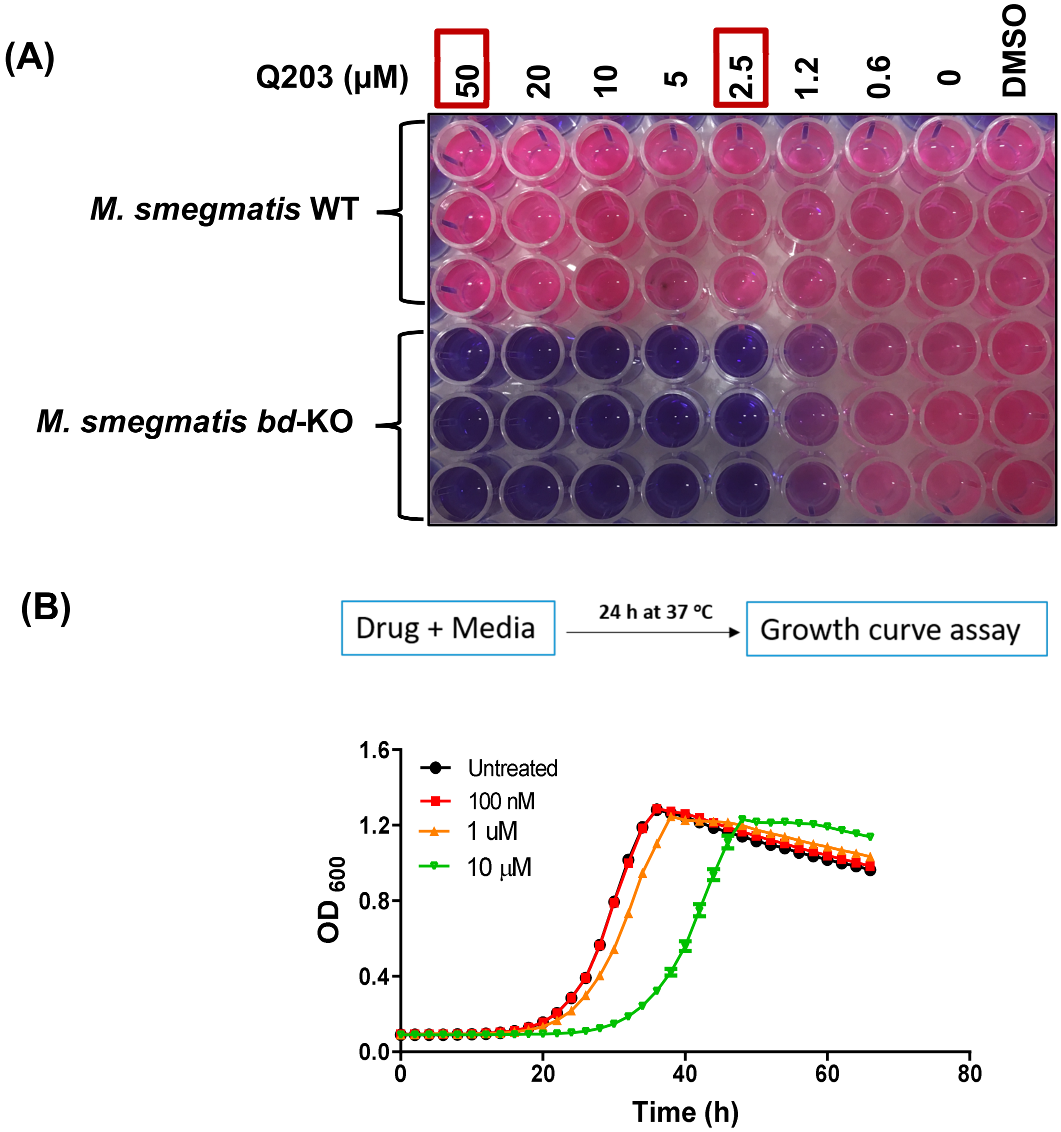

(C)

Q203 / Time (h)	Untreated	100 nM	1 µM	10 µM
Lag phase	14±0.3	14±0.1	14.3±0.2	25±0.3
Doubling time (T_d)	4.2±0.2	4.2±0.1	4.45±0.2	4.3±0.1

Figure S1. (**A**) Estimation of Minimal Inhibitory Concentration (MIC_{90}) of *M. smegmatis* WT and *bd*-KO using resazurin reduction assay. Cultures were treated with 2-fold serial dilution of Q203 for 48 hours followed by addition of alamar blue solution for 24 hours. Estimated MIC for *M.*

smegmatis WT and *bd*-KO are marked with red boxes. (**B**) Growth curve analysis of *M. smegmatis bd*-KO in 7H9 complete media containing Q203 which have been pre-incubated for 24 hours before the addition of cells. (**C**) Growth parameters calculated based on the growth curve obtained indicate no effect of Q203 degradation as extended lag phase in presence of 10 µM (4X MIC) Q203 is still apparent.

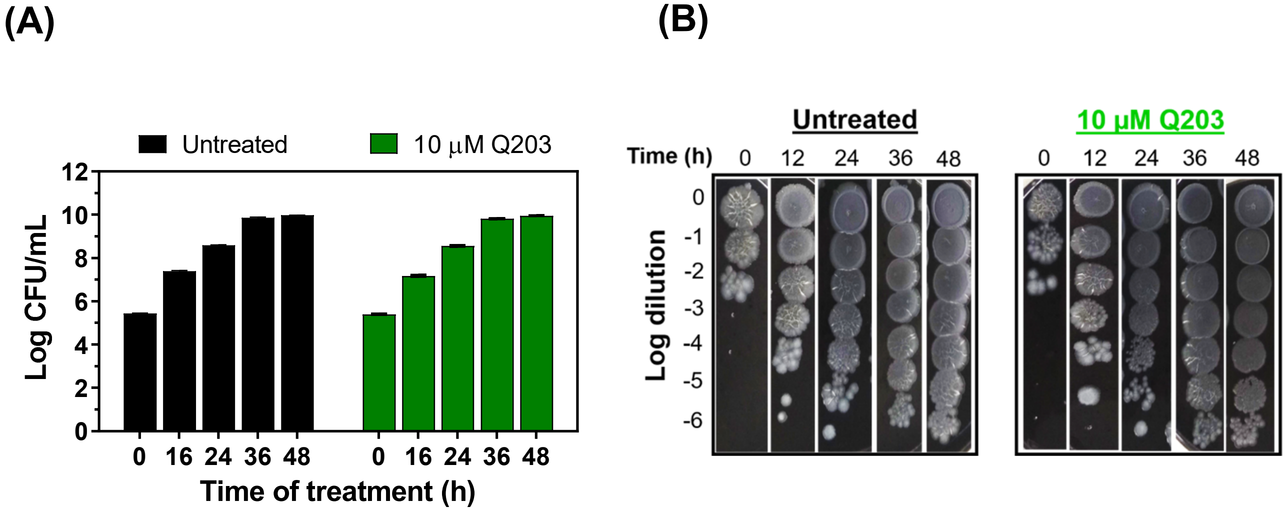

Figure S2.(**A**) Quantification of surviving *M. smegmatis* WT after exposure to 10 µM Q203 for indicated time. CFU/mL were counted on agar plates after three days of incubation at 37 °C. Error bars represent SDs obtained from two independent experiments, each done in triplicate. CFU, colony forming unit and note the log scale of *y* axis. (**B**) Representative dilution series of *M. smegmatis* WT after exposure to 10 µM Q203 for indicated time. Each spot represent 5 µL of aliquot.

(A)

Sequences used for pairwise alignment	Sequence identity (%)	Sequence similarity (%)	Gaps (%)
cydA vs. MSMEG_5584	37.6	37.6	52.1
cydB vs. MSMEG_5585	33.6	33.6	56.7
**CydA vs. MSMEG_5584**	9.8	15.1	63.9
**CydB vs. MSMEG_5585**	1.6	2.6	93.7

(B)

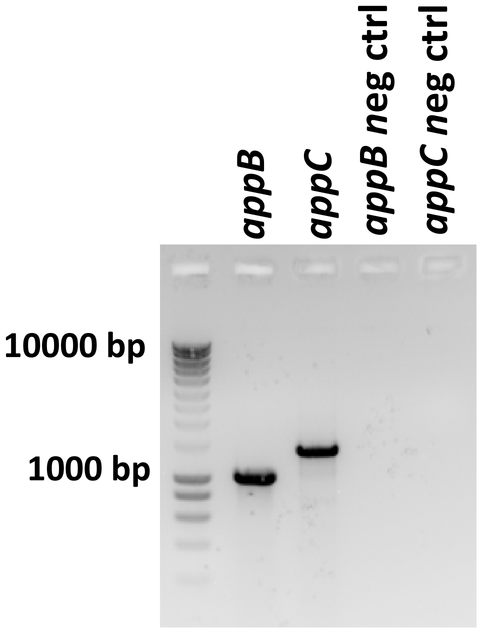

(C)

Sequences used for pairwise alignment	Sequence identity (%)	Sequence similarity	Gaps (%)
cydA vs. appC	49.0	49.0	35.0
cydB vs. appB	50.1	50.1	35.4
**CydA vs. AppC**	26.2	41.2	18.2
**CydB vs. AppB**	22.1	32.8	34.5

Figure S3. (**A**) Pairwise sequence alignment between MSMEG_5584 and *cydA*/CydA as well as between MSMEG_5585 and *cydB*/CydB. (**B**) Validation of the presence of *appB* and *appC* in *M.*

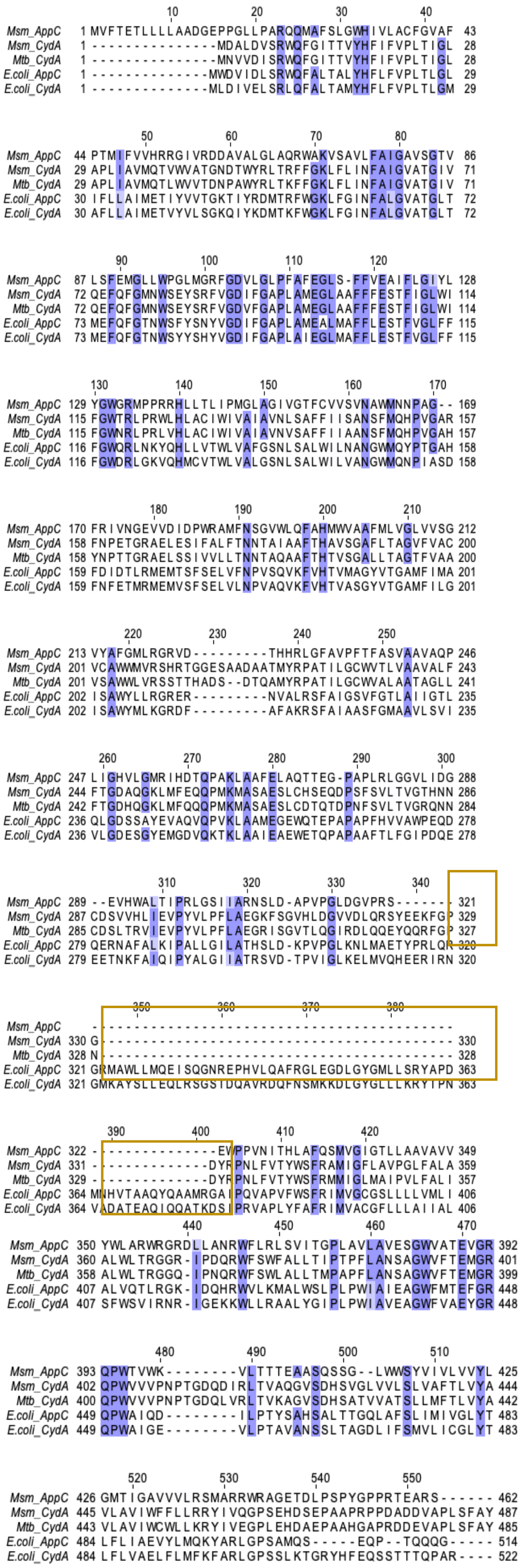

Figure S4. Multiple sequence alignment of complete *M. smegmatis* (Msm) AppC, Msm CydA, *M. tuberculosis* (Mtb) CydA, E. coli AppC and CydA. Alignment was performed using Clustal Omega, via the online tool Jalview. Protein sequences were derived from the UniProt database. Aligned residues with >99% identity were colored, using the BLOSUM62 color scheme. Absence of insert of ~60 residues in AppC is indicated by brown box.

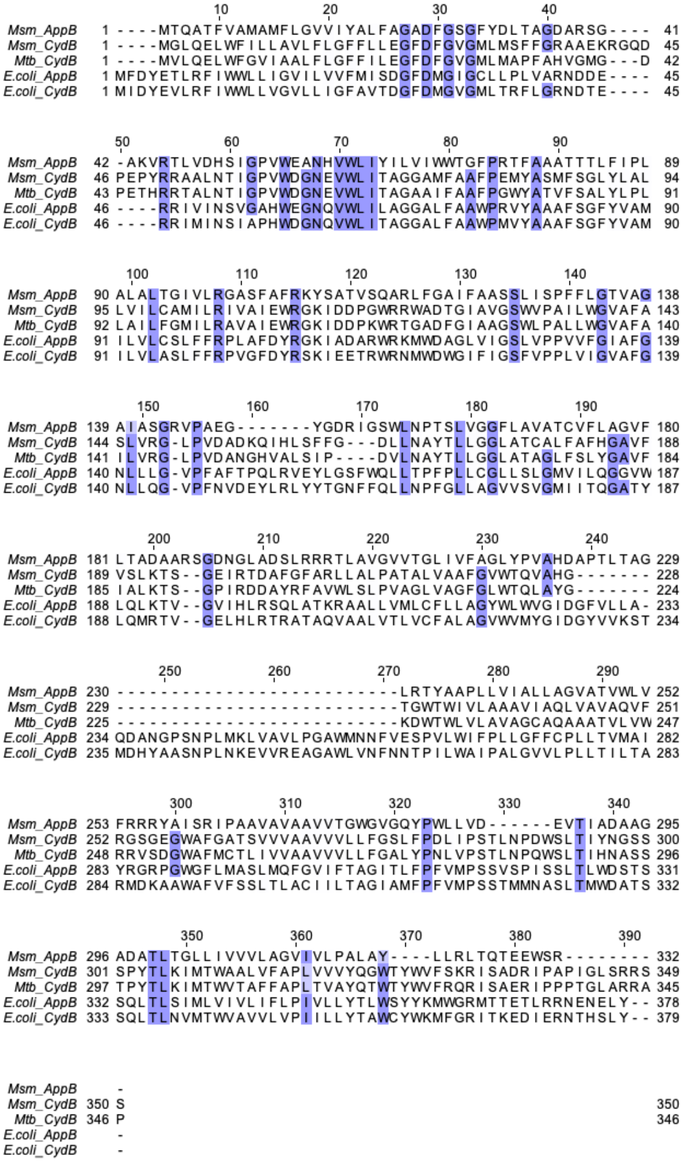

Figure S5. Multiple sequence alignment of complete *M. smegmatis* (Msm) AppB, Msm CydB, *M. tuberculosis* (Mtb) CydB, E. coli AppB and CydB. Alignment was performed using Clustal Omega, via the online tool Jalview. Protein sequences were derived from the UniProt database. Aligned residues with >99% identity were colored, using the BLOSUM62 color scheme.

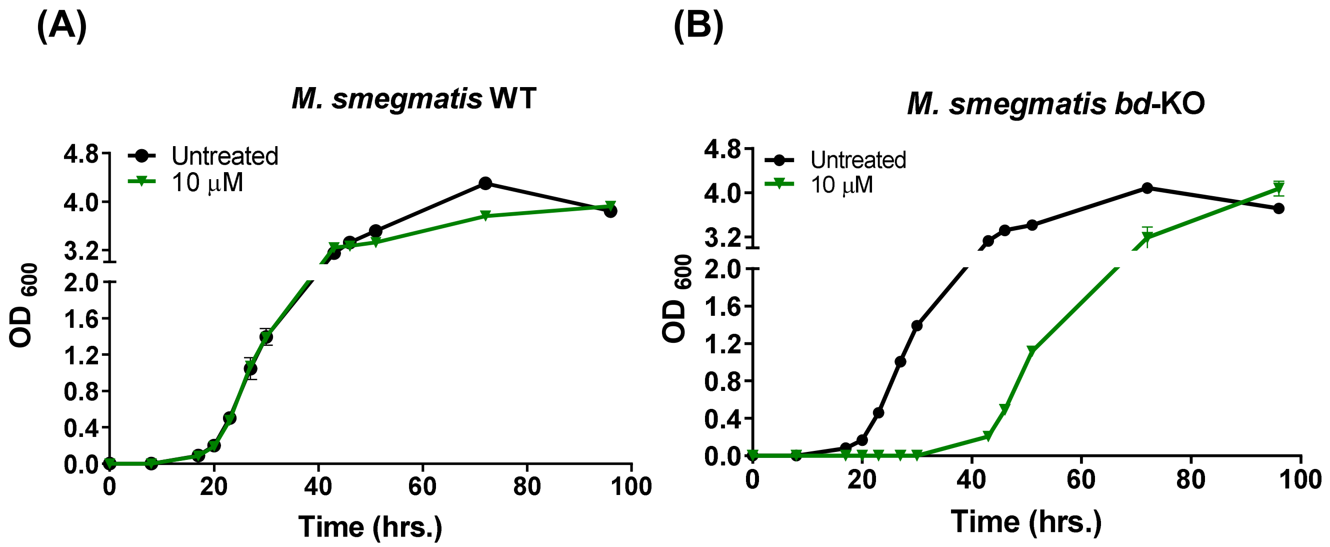

Figure S6. Growth curve analysis of **(A)** *M. smegmatis* WT and **(B)** *M. smegmatis bd*-KO in 7H9 complete media containing Q203 in 50 mL flask. Mean±SD of two biologically independent cultures are plotted.
